# P33 A female with a drooping eyelid and positive autoantibodies: mystery solved?

**DOI:** 10.1093/rap/rkab068.032

**Published:** 2021-10-19

**Authors:** Mark Ford, Lavanya Rajagopala, R Bhatt, H Mudhar, S Venkatachalam

**Affiliations:** The Royal Wolverhampton NHS Trust, Wolverhampton, United Kingdom

## Abstract

**Case report - Introduction:**

The presence of autoantibodies in IgG4-related disease (IgG4-RD) can lead to confusion and we present a case to highlight the diagnostic difficulty in this condition.

**Case report - Case description:**

A 63-year-old Indian female presented with progressive ptosis and swelling of the left eyelid for 2 months. She was referred to the stroke clinic where she was found to have left ptosis with associated swelling. An MRI of the orbit revealed an enlarged left lacrimal gland and mucosal thickening in paranasal sinuses. Her ESR was 20, CRP 2, 15% peripheral eosinophils and IgG 17.55 g/L (6-16). A referral was made to rheumatology due to an abnormal immunological profile: cANCA +, MPO ANCA positive 32 IU/ml (0-3.5) and a strongly positive anti-Ro antibody >600 U/ml (0-10). Differential diagnosis considered: ANCA associated vasculitis (AAV), Primary Sjögren’s syndrome (SS), and IgG4-related orbital disease. She was assessed by the ENT surgeon and there was no sinusitis or evidence of vasculitis. Chest X-ray and CT scan of chest were normal.

A left lacrimal gland biopsy revealed reactive lymphoid hyperplasia with numerous plasma cells and eosinophils. Immunohistochemistry revealed over 50% of plasma cells to be IgG4-positive (approximately 100/HPF) suggestive of IgG4-RD. There was no evidence of granuloma formation or malignancy. Serum IgG4 was raised 6.82 g/l (<1.3). A review by ophthalmology revealed no objective evidence of dry eyes. She was treated as IgG4-RD and demonstrated a marked improvement following treatment with oral steroids and azathioprine.

**Case report - Discussion:**

IgG4-RD is an immune-mediated systemic fibro inflammatory disease characterised by tumefactive lesions with abundant IgG4-positive plasma cells infiltrating into multiple organs. Isolated IgG4-related ophthalmic disease is present in 23% of patients with IgG4-RD, in whom the lacrimal gland is the most commonly involved structure, followed by orbital fat and extraocular muscles. IgG4-related ophthalmic disease is rare and we need to exclude commoner causes of orbital inflammation (such as thyroid ophthalmopathy, granulomatosis with poly-angiitis, sarcoidosis, and tuberculosis) or malignancy.

**Case report - Key learning points:**

The diagnosis of IgG4-RD is often challenging. It is well recognised that small vessel vasculitis can increase the number of IgG4-positive plasma cells in tissue biopsies. Additionally, literature reveals biopsy-proven IgG4-RD can have ANCA positivity with and without concomitant presence of ANCA-associated vasculitis and there are case reports of patients who fulfil criteria for both Sjogren’s syndrome and IgG4-RD. As such, diagnosis requires careful correlation of clinical, radiologic and histological features due to the overlapping features in differential diagnoses.

